# Metabolic Analysis Using HR-MAS in Prostate Tissue to Predict Biochemical Recurrence in Prostate Cancer

**DOI:** 10.5152/tud.2026.25102

**Published:** 2026-10-13

**Authors:** Jorge Panach-Navarrete, Vannina González-Marrachelli, José Manuel Morales-Tatay, Francisco García-Morata, María Ángeles Sales-Maicas, Daniel Monleón-Salvado, José María Martínez Jabaloyas

**Affiliations:** 1 Department of Urology, University Clinic Hospital of Valencia, INCLIVA, Health Research Institute, University of Valencia Faculty of Medicine and Odontology, Valencia, Spain; 2 Department of Physiology, University of Valencia Faculty of Medicine, Valencia, Spain; 3 Institute for Biomedical Research, Hospital Clinic of Valencia (INCLIVA), Valencia, Spain; 4 Department of Pathology, University of Valencia Faculty of Medicine and Odontology, Valencia, Spain; 5 Research Unit, INCLIVA Health Research Institute, Valencia, Spain; 6 Department of Pathology, University Clinic Hospital of Valencia, INCLIVA, Health Research Institute, University of Valencia Faculty of Medicine and Odontology, Valencia, Spain; 7 Department of Metabolomic, University of Valencia, Valencia, Spain

**Keywords:** Metabolomics, prostate cancer, biomarkers, biochemical recurrence

## Abstract

**Objective:**

In this study, nuclear magnetic resonance (NMR) spectroscopy was used in prostate tissue to gather novel data on biomarkers that could predict biochemical recurrence in prostate cancer.

**Methods:**

Prostate tissue cores were collected from individuals who presented to our institution for transrectal biopsy. After acquiring all the spectra using a Bruker Avance III DRX 600 spectrometer, tissue specimens were subjected to routine histology to confirm the presence or absence of prostate cancer. Univariate and multivariate analyses with metabolic and clinical variables were performed to predict biochemical recurrence in men treated for prostate cancer.

**Results:**

Data were collected from 70 patients treated for prostate cancer, 16 of whom developed biochemical recurrence within 5 years of treatment, with an average time to diagnosed recurrence of 25.68 ± 15.39 months. Metabolites detected in tissue, such as O-phosphoethanolamine, ethanol, succinate, or ascorbate, together with ISUP grades of 4 and 5, formed a predictive model for biochemical recurrence with an area under the curve (AUC) of 0.88, a specificity of 91%, and a negative predictive value (NPV) of 88%.

**Conclusion:**

In prostate cancer patients who have undergone treatment, metabolomic evaluation through HR-MAS can serve as a predictor of biochemical recurrence. Metabolites such as O-phosphoethanolamine, ethanol, succinate, ascorbate, and cysteine are useful in this context. Adding another clinical variable, such as ISUP grades 4 and 5, in prostate histology to these metabolites forms a predictive model capable of predicting biochemical recurrence in prostate cancer.

**Main Points**

- HR-MAS NMR–based metabolomic profiling of prostate biopsy tissue can predict biochemical recurrence after curative treatment for prostate cancer.
- A specific tissue metabolomic signature—including O-phosphoethanolamine, succinate, ascorbate, cysteine, ethanol, glycerol, uridine, and formate—was associated with future biochemical recurrence.
- Metabolite-only predictive models outperformed clinical variables alone in predicting biochemical recurrence (AUC: 0.87 vs 0.66).
- Combining metabolomic biomarkers with histopathological grading (ISUP grades 4 and 5) achieved the highest predictive accuracy (AUC: 0.88, specificity: 91%, and negative predictive value: 88%).
- Biopsy-based tissue metabolomics represents a clinically translatable tool for early risk stratification and personalized follow-up in prostate cancer patients.

## Introduction

Globally, prostate cancer continues to rank among the most frequent malignancies affecting men. It represents 11.7% of all newly diagnosed cancers, with rates of 19% in developed nations and 5.3% in developing regions.^1^ Treatment and follow-up management for this neoplasia vary greatly depending on multiple clinical variables: patient age, serum prostate-specific antigen (PSA) level, local or systemic disease involvement, and patient preferences.^2^ One phase of the disease is known as biochemical recurrence, which usually precedes clinical recurrence by years. Biochemical recurrence is defined as 2 consecutive test results with serum PSA above 0.2 ng/mL after radical prostatectomy or a serum PSA level greater than 2 ng/mL above the nadir PSA after radiotherapy, regardless of whether or not hormone therapy has been administered.^3^

Due to the growing interest in obtaining non-invasive diagnostic tools useful in prostate cancer management algorithms, a large number of biomarkers from different scientific disciplines have been described in recent years.^4^ Metabolomics, within the family of omics sciences, utilizes nuclear magnetic resonance (NMR) to investigate metabolic pathways linked to disease, analyzing both tissue samples and biofluids, such as saliva or urine, collected through non-invasive methods. Indeed, metabolomics has revealed various metabolic pathways involved in prostate cancer development that could be useful for disease diagnosis, the most relevant of which involve metabolites such as citrate, glycero-3-phosphocholine, glycine, carnitine, and O-phosphocholine.^5,6^

The objective of this research was to apply NMR spectroscopy on prostate tissue to detect candidate biomarkers linked to biochemical recurrence in prostate cancer patients who had undergone treatment.

## Material and Methods

### Study Design and Inclusion Criteria

This observational, prospective study comprised consecutive men undergoing clinically indicated transrectal prostate biopsy at our center during a 2-year period. This study was conducted between January 1, 2015, and December 31, 2016. These subjects were invited to participate in a study collecting prostate tissue specimens for subsequent metabolic analysis using HR-MAS. The only inclusion criteria were age over 18 years and signed informed consent to participate in the study. For the present study, we selected the subgroup of patients diagnosed with prostate cancer subsequently treated with radical prostatectomy or curative-intent radiotherapy. The sample was stratified into those with vs without biochemical recurrence. Figure 1 shows the flow chart of patient selection. Of the 201 patients included for tissue specimen collection, 70 had received treatment with surgery or radiotherapy.

Written informed consent was obtained from all participants. This study was approved by the Ethics Committee of INCLIVA (Instituto de Investigación Sanitaria, Health Research Institute, Valencia, Spain) (Approval no: 12-2014; Date: December 1, 2014).

**Figure 1.**
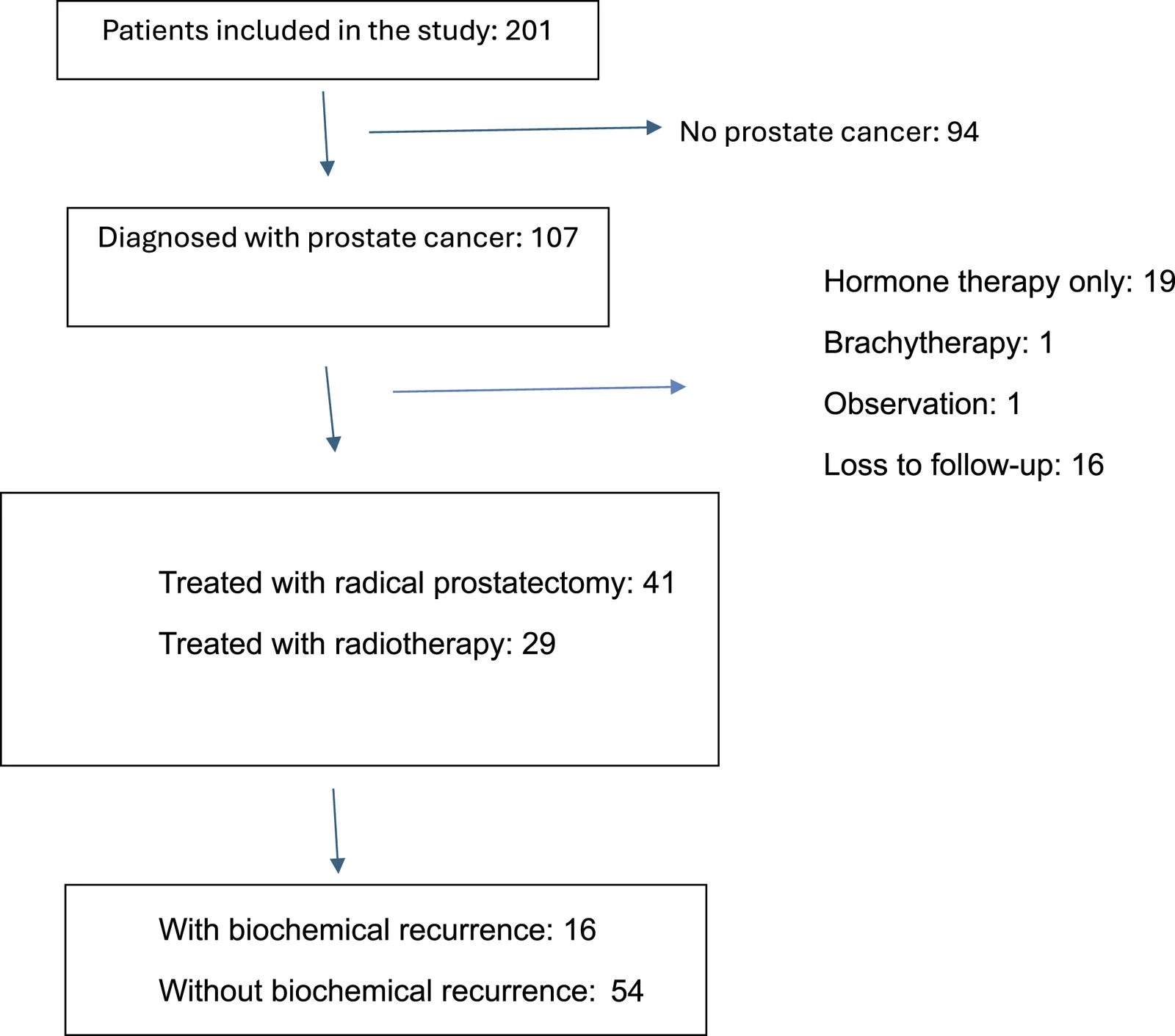
Flow chart of patient selection for the study.

### Specimen Collection

The prostate biopsy performed was a systematic transrectal biopsy, not a fusion biopsy. For each patient, 10 cores were obtained for conventional histological evaluation (referred to as “standard biopsy”), along with 1 additional core reserved for metabolomic assessment and subsequent histological examination (“HR-MAS core”). The HR-MAS samples were delivered on the day of collection to the laboratory, where they were frozen at −80°C until processing and analysis. After spectroscopy, the HR-MAS core was fixed in formalin and submitted to the Pathological Anatomy Department for histological assessment. At the same time, the 10 standard biopsy cores designated for routine histology were immediately placed in formalin after retrieval and transported for pathological evaluation. A diagnosis of prostate cancer was established when either the HR-MAS core or the standard biopsy cores revealed prostatic carcinoma upon histological review. The study database was updated prospectively with each case. Baseline clinical data were recorded at inclusion, whereas pathological findings were entered 1 month after specimen collection. For patients diagnosed with prostate cancer, therapeutic management (or loss to follow-up) was documented, along with the occurrence of biochemical recurrence during the 5-year follow-up period, as defined in the Introduction. Of all the HR‐MAS cores submitted to metabolic and subsequently routine histological study, 56 (27.8%) were positive for carcinoma. The relationship of these carcinoma-positive cores with metabolite levels can be found in another publication from our research group.^5^

### Specimen Processing for Metabolic Profiling Using HR-MAS

HR-MAS core analyses were performed with a 4-mm high-resolution magic angle spinning probe for semi-solids. For sample preparation, the entire frozen specimen available (1-12 mg) was placed into a 50-µL rotor together with 25 µL of D_2_O. All handling and rotor loading procedures were carried out under cold conditions, using liquid nitrogen to avoid thawing or sample degradation. The preparation process required less than 5 minutes. Spectra acquisition was conducted at a stable temperature of 277 K inside the rotor.

### Prostate Tissue Specimens

All spectra were acquired on a Bruker Avance III DRX 600 spectrometer (Bruker BioSpin GmbH, Rheinstetten, Germany) operating at a ^1^H frequency of 600.13 MHz. The spectrometer is equipped with an HR-MAS triple resonance probe for ^1^H–^13^C–^15^N nuclei, with a set of 4-mm rotors, and the probe’s magnetic field gradient is aligned with the magic angle axis. The spin rate of the specimens was kept at 5000 Hz to keep the rotational sidebands out of the acquisition window. To minimize the effects of tissue degradation with temperature, which would alter the metabolic composition of the specimen, all spectra were acquired at a temperature of 277 K. Before performing the corresponding measurements on the specimens, the possible difference between the internal temperature of the rotor and the nominal 1 was corrected by calibration with a 100% methanol sample. For all specimens, a 15-minute single-pulse water presaturation experiment was run, with a 2.27-second acquisition time, 256 repetitions, 32 768 acquired points, and a 1-second relaxation time. Water presaturation was used for 1 second throughout the recycle time for solvent signal suppression. The spectral width for all spectra was 8000 Hz (12 ppm) for the ^1^H spectrum. Before applying the Fourier transform to the data, the FID (free induction decay) was multiplied by an exponential function of 1 Hz linewidth.

Detailed protocols for NMR acquisition and processing are available in Supplementary Material 1.

### Statistical Analysis

Statistical evaluations were conducted using SPSS Statistics, version 20 (IBM Corp., Armonk, NY, USA). Comparisons were made between patients with and without biochemical recurrence after treatment. The clinical variables considered included age, PSA, PSA density, prostate volume, findings on digital rectal examination, tumor suspicion, and ISUP grade. For patients treated with radiotherapy, prostate biopsy information was used, while for those undergoing surgery, prostatectomy specimens were analyzed. Univariate analyses (Student’s *t* test, chi-squared test, and median test) were first applied, followed by multivariate modeling (binary logistic regression) whenever univariate results suggested potential significance (*P* < .3). The outcome variable in these comparisons was biochemical recurrence, with significance set at *P* < .05. Multivariate assessment of metabolomic profiles was conducted in MATLAB R2019b (MathWorks, Natick, MA, USA) using routines developed in-house and the PLS Toolbox (Eigenvector Research Inc., Manson, WA, USA). Advanced statistical methods, including principal component analysis (PCA) for pattern recognition and partial least squares discriminant analysis (PLS-DA) for class separation, were employed to construct predictive models. Finally, mixed models integrating clinical variables with metabolomic features were developed, and their predictive ability was validated through receiver operating characteristic curves and corresponding component analyses.

This study did not use AI and/or LLM tools during the preparation of this manuscript.

## Results

### Specimen Characteristics

A total of 70 patients received treatment and were included in the study, of whom 16 experienced biochemical recurrence, with an average time to diagnosed recurrence of 25.68 ± 15.39 months. Comparative analysis of the clinical variables in each study group (cases with vs without biochemical recurrence) is given in Table 1. The only variables with a tendency toward statistical significance were the PSA ratio (0.10 in cases with recurrence vs 0.12 in cases without, *P* = .20), suspicious digital rectal examination (56.2% in the recurrence group vs 32.9% in the non-recurrence group, *P* < .08), and ultrasound suspicion of tumor (75% in the recurrence group vs 57.4% in the non-recurrence group, *P* = .21).

**Table 1.** Univariate Analysis

Variable	Recurrence (16 Patients)	No Recurrence (54 Patients)	*P*
Age in years (average ± SD)	67.53 ± 9.69	68.76 ± 8.70	.49^a^
PSA in ng/mL (median, range)	11.5 (7-1446)	9 (3-237)	.78^b^
PSA density (median, range)	0.35 (0.13-3.20)	0.26 (0.06-24.80)	.95^b^
PSA ratio	0.10 ± 0.08	0.12 ± 0.06	.20^a^
Vol in cc (average ± SD)	34.92 ± 13.70	39.19 ± 19.32	.43^a^
Suspicious digital rectal examination (n [%])	9 (56.2%)	17 (32.9%)	<.08^c^
Suspicion on ultrasound (n [%])	12 (75%)	31 (57.4%)	.21^c^
ISUP grades 4 and 5 in biopsy/prostatectomy specimen	12 (75%)	16 (29.6%)	.04^a^

Note: Differences in clinical characteristics between the 2 study groups (with vs without biochemical recurrence).

ISUP, International Society of Urological Pathology; PSA, prostate-specific antigen; SD, standard deviation; Vol, volume.

a Mann–Whitney *U* test.

b Median test.

c Chi-squared test.

The subsequent multivariate analysis showed that patients with unfavorable histology in biopsy or prostatectomy specimen (ISUP grades 4 and 5) were 2.5-fold more likely to develop biochemical recurrence than those with ISUP grades 1-3 (Table 2). No other clinical variable was related to the development of biochemical recurrence.

**Table 2.** Multivariate Analysis

Variable	OR	95% CI	*P* Value^a^
PSA ratio	0.05	0.01-263.43	.49
Suspicious digital rectal examination	2.35	0.60-9.21	.21
Suspicion on ultrasound	1.14	0.24-5.24	.86
ISUP grades 4 and 5	2.5	1.25-3.45	.04

Note: Binary logistic regression including clinical variables with a tendency toward statistically significant differences in the previous univariate analysis (cases with and without biochemical recurrence). CI, confidence interval; ISUP, International Society of Urological Pathology; OR, odds ratio; PSA, prostate-specific antigen.

a Binary logistic regression.

### HR-MAS Determination of the Metabolic Fingerprint of Prostate Cancer with Future Biochemical Recurrence in Transrectal Biopsy–Obtained Cores

The average biopsy core volume measured by metabolomics was 4.85 mg, with a minimum specimen size of 0.9 mg and a maximum of 12.40 mg. Although the specimen volume was limited, the spectra showed favorable signal-to-noise ratios and clear multiplicity resolution in the majority of peaks. In total, 80 spectral regions were quantified, corresponding to 70 identified metabolites and 10 unknown compounds. Supplementary Table 1 shows the average and standard deviation of the spectra in tissue from cases with and without recurrence.

### Unsupervised Analysis: PCA, the Ability of HR-MAS Metabolomics to Build a Model to Assess Biochemical Recurrence in Treated Patients

PCA showed no spontaneous separation in the distribution of the 2 groups (Figure 2). Cases with recurrence, although mostly clustered in the 2 right quadrants, also cluster heterogeneously throughout the scatter plot, and the same is observed for the cases without biochemical recurrence.

**Figure 2.**
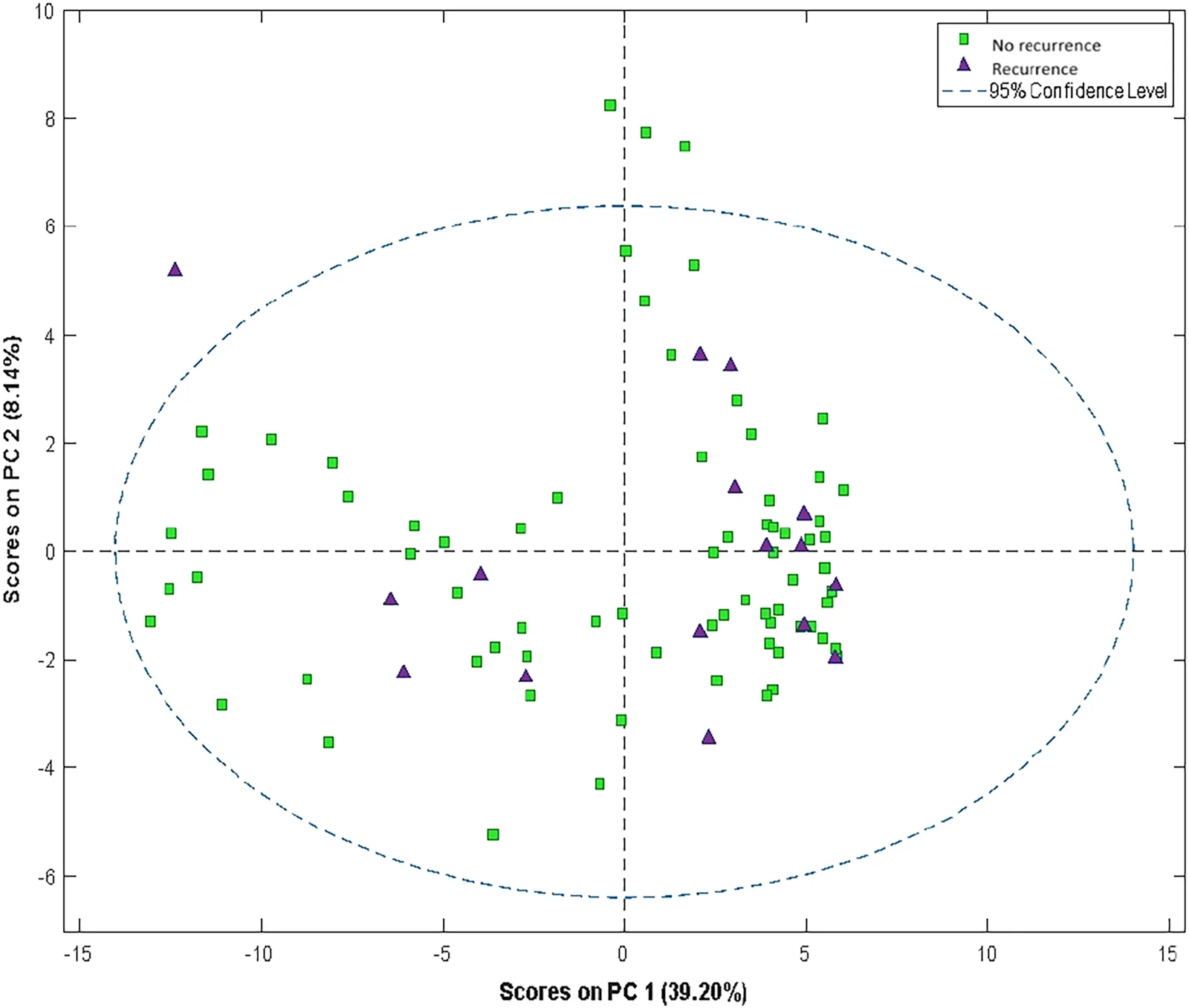
Scatter plot obtained from PCA of the ^1^H-HR-MAS PRESAT spectra of prostate tissue specimens in treated cancer patients, showing cases with biochemical recurrence (purple triangles) and cases without biochemical recurrence (green squares). PCA, principal component analysis.

### Supervised Analysis: PLS-DA, the Capability of HR-MAS Metabolomics to Build a Model to Assess Biochemical Recurrence in Treated Patients

To examine the different distributions in the diagram according to biochemical recurrence, we performed supervised analysis, pre-establishing the groups and checking for any tendency for groups to separate. The same cases as for the PCA were included. The scatter plot in Figure 3 shows a (non-substantial) separation between groups. Most recurrent cases were clustered in the upper right quadrant, although a proportion also appeared in other quadrants. Cases without recurrence were distributed heterogeneously throughout the ellipse, representing a 95% confidence interval. Therefore, a certain tendency toward separation between groups was observed in the PLS-DA, although a “gray zone” with overlap between cases with and without biochemical recurrence can also be identified.

**Figure 3.**
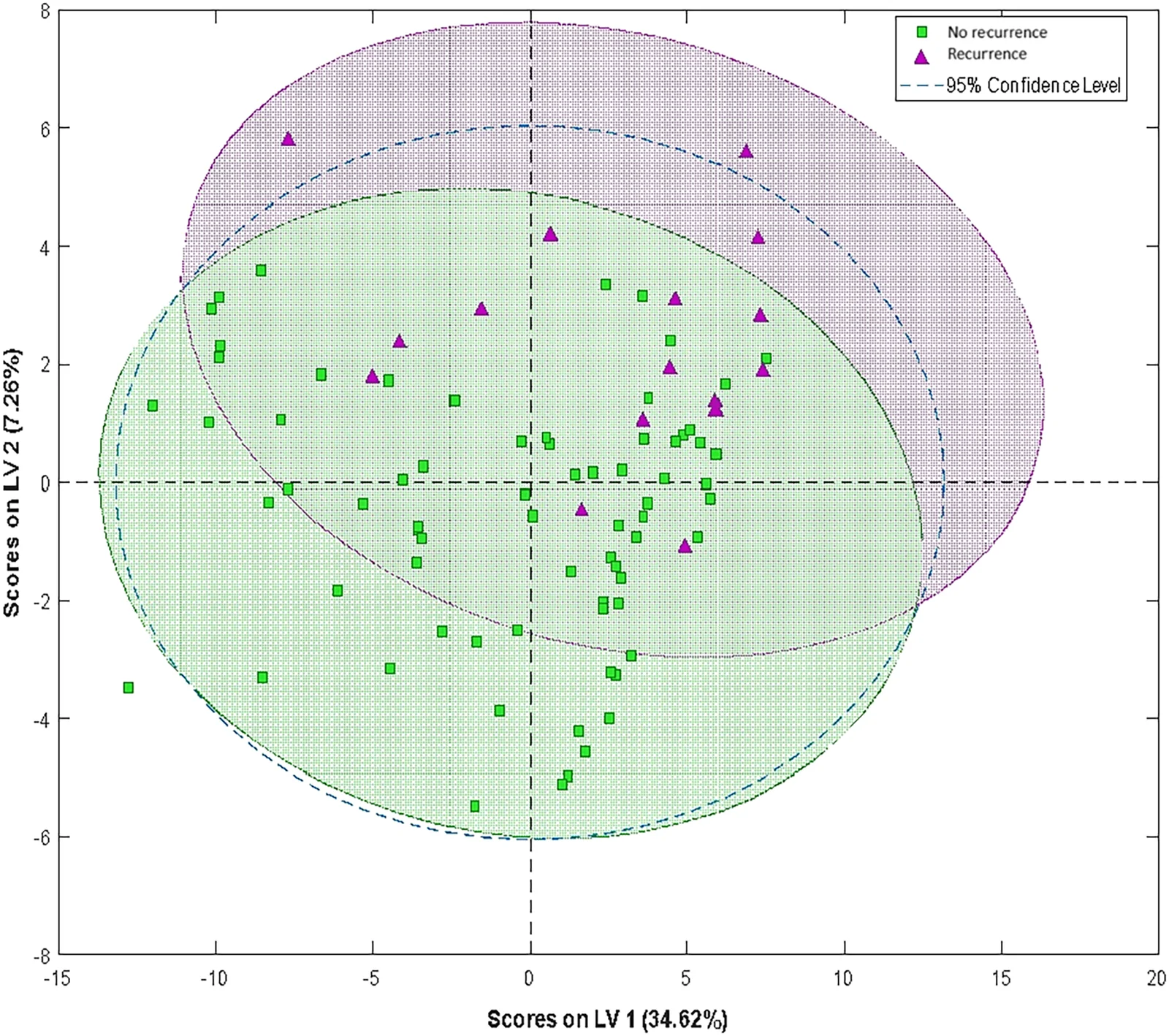
Scatter plot obtained from PLS-DA of the ^1^H-HR-MAS PRESAT spectra of prostate tissue specimens grouped into cases with (purple triangles) and without biochemical recurrence (green squares). The colored ellipses represent a 95% confidence interval for that group. PLS-DA, partial least squares discriminant analysis.

Cross-validation of data was performed for the multivariate PLS-DA statistical model discriminating between cases with and without biochemical recurrence to validate and evaluate the results obtained. The validation result is graphically represented with a ROC curve, shown in Figure 4. The results obtained show that the cross-validation curve (in green) differs from the line corresponding to the estimated data (blue). The area under the curve (AUC) value was 0.45, classifying the ROC curve as poor. The confusion matrix is not shown given the poor prediction of the model, as reflected by the AUC value.

**Figure 4.**
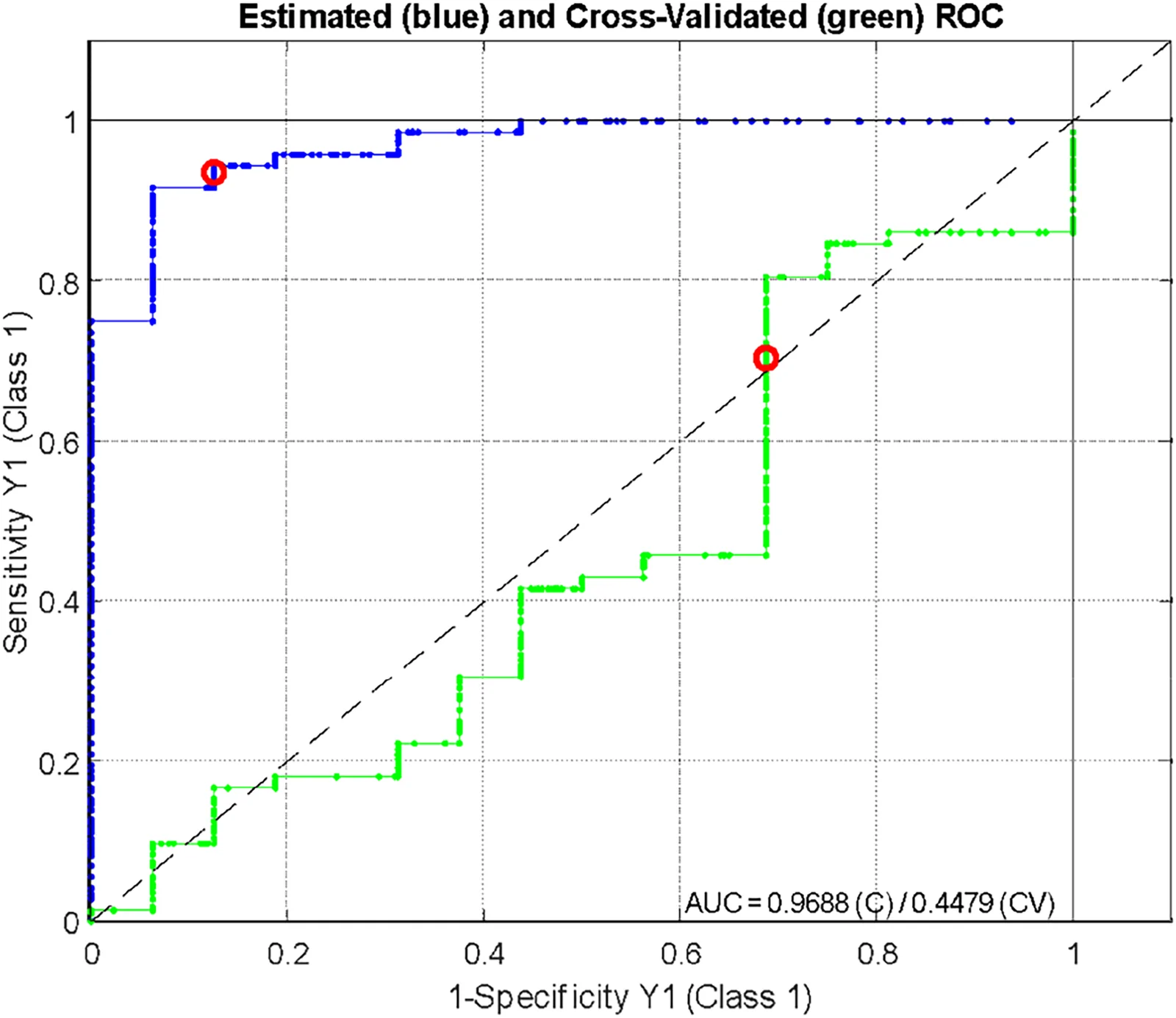
ROC curve obtained from cross-validation of the PLS-DA model discriminating between cases with and without biochemical recurrence, with cases involving subjects who received some type of treatment for prostate cancer. The blue line corresponds to the treated data, and the green line corresponds to the cross-validation. PLS-DA, partial least squares discriminant analysis; ROC, receiver operating characteristic.

### Metabolic Profile and Metabolic Pathways Related to Biochemical Recurrence

After performing PLS-DA, the metabolites exerting the greatest influence (VIP > 1) were identified, as shown in Supplementary Table 2. The most relevant alterations involved pathways related to the Krebs cycle, amino acid metabolism, and glucose utilization. Logistic regression was subsequently applied to assess the effect of selected metabolic variables on the binary outcome of biochemical relapse. A total of 9 metabolites with VIP scores above 1 contributed to group discrimination: formate, glycerol, uridine, ethanolamine, cysteine, ascorbate, succinate, ethanol, and O-phosphoethanolamine (Supplementary Figure 2).

Univariate analysis was performed for each of the 9 selected metabolites. Normal distribution of concentrations was verified using the Kolmogorov–Smirnov test (*P* > .05), and the averages of the 2 groups were compared using the Student’s *t* test. The results of this analysis are included in Supplementary Table 3. All the selected metabolites except ethanolamine and glycerol showed a tendency toward statistical significance between groups (*P* < .3). However, given the weight of the 9 metabolites in the PLS-DA, verified by the VIP score > 1, all were considered necessary for correct discrimination between the metabolomic profiles of patients with and without biochemical relapse and were included in the subsequent multivariate analysis (Supplementary Table 4).

### Models with Clinical Variables and Metabolic Profile

Univariate and multivariate analyses of clinical factors associated with prostate disease are presented in Supplementary Tables 5 and 6. Among the clinical variables studied, only ISUP grades 4 and 5 were independently linked to biochemical recurrence. A multivariate model was then constructed, incorporating both the most relevant metabolites identified through PLS-DA and the clinical variables that demonstrated borderline significance in the univariate analysis (*P* < .3). The outcome of this combined model is detailed in Table 3.

**Table 3.** Multivariate Analysis

Metabolite	OR	95% CI	*P* Value^a^
O-phosphoethanolamine	1.11	0.98-1.26	.09
Ethanol	1.05	0.89-1.23	.55
Succinate	5.29	0.80-34.76	.08
Ascorbate	1.80	1.17-2.76	.007
Cysteine	1.26	0.57-0.97	.03
Ethanolamine	1.24	0.91-1.68	.16
Uridine	0.63	0.44-0.91	.01
Glycerol	0.91	0.85-0.98	.02
Formate	0.01	0.01-0.21	.01
PSA ratio	0.01	0-21.20	.25
Suspicious digital rectal examination	2.82	0.36-21.96	.32
Suspicion on ultrasound	0.28	0.03-2.55	.26
ISUP grades 4 and 5	23	1.20-3.48	.04

Note: Joint binary logistic regression including clinical variables and metabolites (cases with and without biochemical recurrence). CI, confidence interval; ISUP, International Society of Urological Pathology; OR, odds ratio; PSA, prostate-specific antigen.

a Binary logistic regression.

Using joint binary logistic regression, 3 predictive models were evaluated: 1 based exclusively on metabolites with VIP > 1, a second including only clinical variables, and a third integrating both sets of features. ROC curves for these models are illustrated in Figure 5, while Table 4 summarizes the AUC values, 95% confidence intervals, and the main quality indicators derived from the curves. The model with the strongest discriminatory power between patients and controls combined the top VIP metabolites (formate, glycerol, uridine, ethanolamine, cysteine, ascorbate, succinate, ethanol, and O-phosphoethanolamine) with selected clinical variables, such as the PSA ratio, suspicious ultrasound findings, abnormal digital rectal examination, and ISUP grades 4 and 5. This integrated model achieved the highest sensitivity and specificity, with a negative predictive value of 88% for identifying biochemical recurrence.

**Figure 5.**
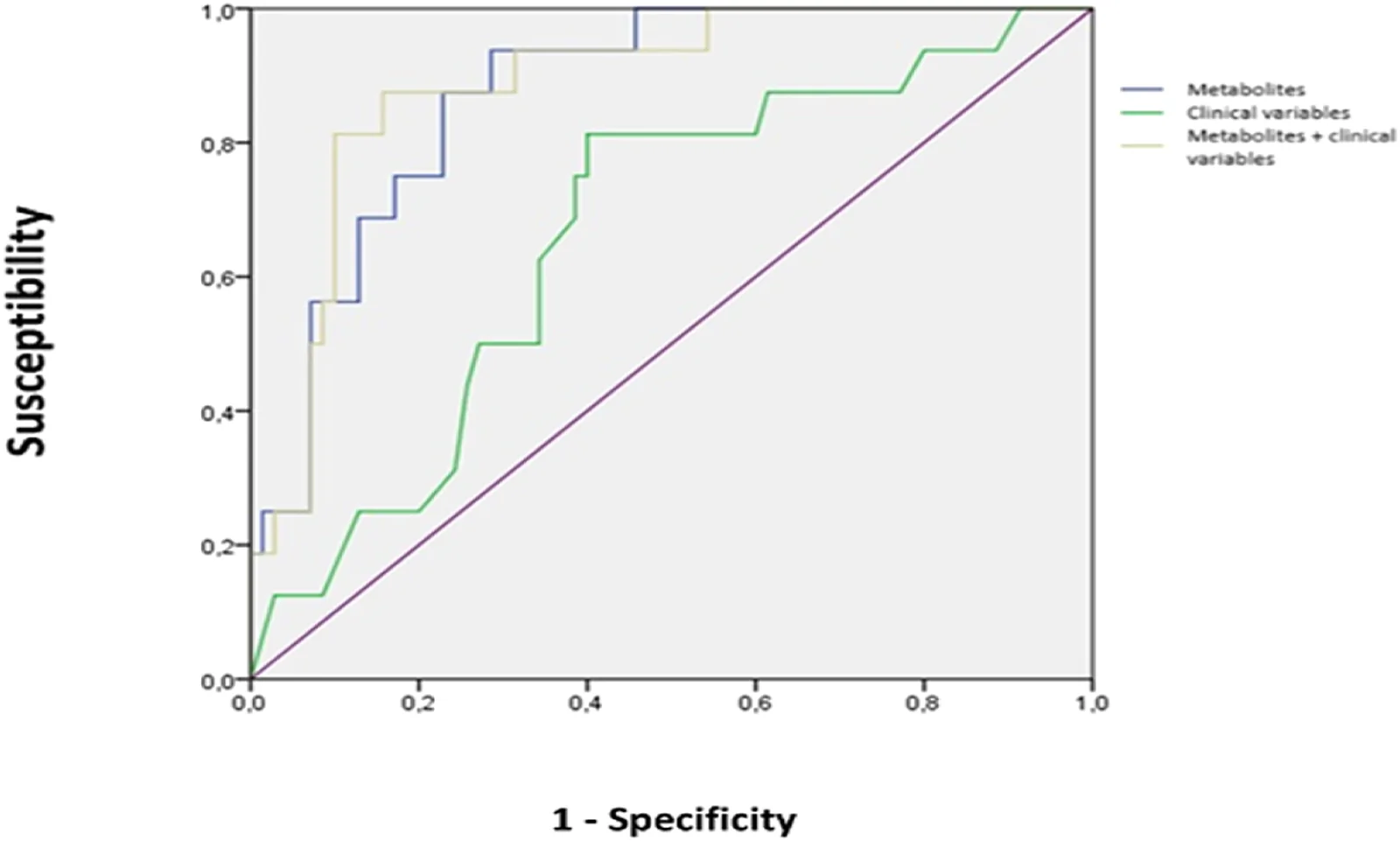
ROC curves obtained from the 3 multivariate models constructed for predicting biochemical recurrence. ROC, receiver operating characteristic.

**Table 4.** Results of the ROC Curves in the 3 Calculated Models

Model	AUC	95% CI—AUC	Sensitivity (%)	Specificity (%)	PPV (%)	NPV (%)
Clinical variables	0.66	0.52-0.80	10	80	20	70
Metabolites	0.87	0.79-0.95	25	93	44	84
Clinical variables and metabolites	0.88	0.80-0.96	50	91	57	88

AUC, area under the curve; CI, confidence interval; NPV, negative predictive value; PPV, positive predictive value; ROC, receiver operating characteristic.

## Discussion

This study highlights the potential of metabolomics to advance the identification of biomarkers in prostate cancer management. Several novel aspects distinguish the present work from prior publications. First, the use of HR-MAS allowed metabolic profiling of tissue specimens while preserving the material for subsequent histological assessment, which can facilitate future investigations that directly relate metabolic alterations to histopathological features in the same sample. Another relevant feature of our study is the inclusion of multiple clinical variables. Whereas previous studies often focused on a single parameter, such as PSA or PSA density,^5,7,8^ we incorporated a broader set of routinely employed clinical indicators for prostate cancer diagnosis and follow-up. By integrating these variables with metabolomic data in mixed models, we provided a translational perspective to what is usually considered a basic research approach. Metabolomic models were first evaluated independently to determine their intrinsic predictive capacity. The subsequent inclusion of histopathological variables reflects a translational, incremental approach rather than a conceptual shift.

HR-MAS profiling was performed on a single systematically obtained biopsy core per patient, with a limited tissue volume. Given the well-known multifocal and heterogeneous nature of prostate cancer, a single small core cannot be assumed to fully represent the entire tumor. Therefore, metabolomic results obtained from HR-MAS should be interpreted as reflecting a localized tissue snapshot that serves as a patient-level surrogate rather than a comprehensive depiction of whole-tumor biology. Any sampling-related heterogeneity would be expected to attenuate metabolic differences and bias results toward the null, potentially underestimating true biological associations. Future studies incorporating multiple cores or precise quantification of tumor content within each HR-MAS specimen may further refine the predictive value of tissue-based metabolomic approaches. Although HR-MAS analysis was performed exclusively on biopsy cores, this approach reflects the tissue sampled at diagnosis and preserves clinical translatability. While larger prostatectomy specimens might yield a broader metabolic representation, current evidence suggests that biopsy-based metabolomic profiles are sufficient to capture clinically relevant signatures associated with prostate cancer outcomes.

Early research on metabolomics in prostate cancer began more than 20 years ago. Cheng and Swanson were among the first to report on metabolic signatures in malignant prostate tissue and to compare them against non-cancerous samples, each contributing 2 landmark publications to the field.^7,9-12^ Nonetheless, our work differs from these early studies on metabolomics in prostate cancer by addressing a specific aspect of this disease: predicting a clinical state (biochemical recurrence). If a differential metabolic fingerprint can be established in men with cancer who experience biochemical recurrence after treatment, these patients could be monitored more closely in clinical practice.

There are various nomograms in clinical practice that use clinical variables to try to predict this stage of the disease.^13^ In our results, the PLS-DA–derived model showed no clear separation of the sample into different groups based on biochemical relapse status. However, by selecting the most influential metabolites, we obtained a model with good discriminatory capacity (AUC of 0.87), proving superior to the 1 comprising clinical variables.

Few works have been published on tissue metabolomics and biochemical recurrence in prostate cancer. In the first study, by Maxeiner in 2010,^14^ 16 consecutive cases of men with biochemical recurrence after radical prostatectomy were compared with 16 cases without recurrence. Via PCA, the authors identified 27 influential regions common to both groups, of which several regions discriminated between the 2 groups, creating a model with an accuracy of 78% to predict biochemical recurrence after surgery. The metabolites implicated in biochemical recurrence were spermine, glutamine, myo-inositol, phosphorylcholine, scyllo-inositol, and glutamate. As a methodology, they used a canonical correlation analysis, studying the bidirectional relationship to discover the correlation between the 2 data sets.^14^ The average follow-up after treatment in their study was 47 months, similar to ours. We acknowledge that a longer follow-up time would have increased the number of biochemical recurrence cases diagnosed. Nonetheless, we consider our results make an important contribution, first taking into account the scarce literature published to date on the subject, and second because using HR-MAS we have identified 9 metabolites capable of creating a model with high specificity and negative predictive value for biochemical recurrence (93% and 84%, respectively). None of our PLS-DA–derived metabolites with a high VIP score coincide with those proposed by Maxeiner in his publication.^14^

Two other publications in the same line are the works of Braadland and Vandergrift.^15,16^ The former collected a sample of 110 patients who underwent radical prostatectomy, of which 50 developed biochemical recurrence. They verified by means of survival analysis that high intratumoral concentrations of spermine and citrate were related to absence of biochemical recurrence, while high (total choline + creatine)/spermine and (total choline + creatine)/citrate ratios were associated with shorter time to recurrence. Furthermore, spermine and the (total choline + creatine)/spermine ratio were independently related to recurrence in multivariate analysis.^15^

In Vandergrift’s study, data were retrospectively collected from 185 patients, from which 365 carcinoma specimens from prostatectomies, 15 specimens corresponding to benign hyperplasia, and 14 benign specimens from prostate biopsies were extracted. The authors attempted to establish a distinct metabolic profile according to tumor aggressiveness (comparing ISUP grades) and studied biochemical recurrence prediction using metabolomics. In addition to confirming elevated concentrations of myo-inositol, glycero-3-phosphocholine, phosphocholine, and valine in specimens with high ISUP grades, they could construct a model to predict recurrence. With an 83% true-positive rate for predicting recurrences, the most influential metabolites were choline, phosphocholine, glycero-3-phosphocholine, and glutamate, whereas high levels of myo-inositol showed a significantly lower risk of recurrence during follow-up.^16^

From the PLS-DA, several metabolic pathways emerged as highly relevant to biochemical recurrence, including amino acid metabolism (e.g., cysteine), glycolytic intermediates such as glycerol, nucleosides such as uridine, and inositol-related compounds such as ascorbate. Cancer cells are known to display increased glycolytic activity to support both energy production and biosynthesis. Although aerobic glycolysis is not exclusive to prostate cancer, enhanced glycolytic activity has been consistently described in advanced disease stages. Key enzymes implicated in this process include GLUT1, HK1, HK2, and PFK2, all of which are under the regulatory influence of the androgen receptor.^17^ In terms of amino acid metabolism, many metabolic pathways serve as a source of carbon and nitrogen in the tricarboxylic acid cycle, producing NADPH, as well as accumulating mitochondrial carbon for maintaining mitochondrial membrane potential and for synthesizing nucleotides, proteins, and lipids.^18^ Finally, uridine confers rigidity to the RNA structure, contributing to stabilization, decoding activity, and the formation of peptide bonds. As measured by epitranscriptomic analysis, it has been proposed as a specific marker of prostate cancer.^19,20^

The main limitations of this work include its single-center design and the relatively small number of cases analyzed. The relatively small number of biochemical recurrence events limits statistical power and may increase the risk of overfitting; therefore, the present findings should be interpreted as exploratory and require validation in larger, independent cohorts. In addition, a follow-up period of 5 years may not be sufficient to fully capture the long-term course of prostate cancer after treatment. Nevertheless, our study contributes valuable information regarding the pathophysiology of the disease and may help inform its clinical management.^21^ Future investigations could validate the localization of relevant biomarkers in tissue and assess their utility through non-invasive approaches, such as urine analysis after spontaneous voiding.^22^ Moreover, the present findings may serve as a basis for future applications, including the development of in vivo MRI strategies. HR-MAS profiling was performed on 1 systematically obtained biopsy core collected at the time of diagnostic biopsy. Given the multifocal nature of prostate cancer and the limited volume of a single core, variable proportions of tumor, benign glands, stroma, and inflammatory components are expected. Because our end point (biochemical recurrence) and prediction models are defined at the patient level, this heterogeneity reflects real-world sampling conditions under which a tissue-based metabolomic tool would be used. Importantly, any inclusion of benign tissue would be expected to attenuate metabolite contrasts and bias results toward the null; thus, the observed discrimination is likely conservative. Nevertheless, we acknowledge that future larger cohorts could evaluate the incremental value of stratifying by tumor content within the HR-MAS core.

In conclusion, in prostate cancer patients who have undergone treatment, metabolomic evaluation through HR-MAS can serve as a predictor of biochemical recurrence. Metabolites such as O-phosphoethanolamine, ethanol, succinate, ascorbate, and cysteine are useful in this context. Adding another clinical variable, such as ISUP grades 4 and 5, in prostate histology to these metabolites forms a predictive model capable of predicting biochemical recurrence in prostate cancer.

## Data Availability

The data that support the findings of this study are available on request from the corresponding author.
